# Establishing a pharmacist–prescriber partnership in publicly funded primary healthcare clinics to optimise antibiotic prescribing in the Western Cape: An exploratory study

**DOI:** 10.4102/safp.v62i1.5090

**Published:** 2020-06-22

**Authors:** Oliver van Hecke, Renier Coetzee

**Affiliations:** 1 Nuffield Department of Primary Care Health Sciences, University of Oxford, Oxford, United Kingdom; 2 School of Pharmacy, Faculty of Natural Science, University of the Western Cape, Cape Town, South Africa

**Keywords:** Antimicrobial stewardship, AMS, primary care, pharmacy, antibiotics

## Abstract

**Background:**

Promoting evidence-based antibiotic prescribing through successful antimicrobial stewardship (AMS) programmes is critical to preserving the effectiveness of antibiotics for common infections in primary care. This requires a coordinated multidisciplinary effort. Such pharmacist–prescriber partnerships have been effective in high-income countries (HICs). Yet, evidence generated in such countries is not always applicable because of different social determinants of health.

**Methods:**

A multidisciplinary workshop was conducted with pharmacists and clinicians (doctors, nurses) on community-based antibiotic stewardship, the purpose of which was to explore how and where such partnerships might work in publicly funded primary care clinics in the greater Cape Metro region.

**Results:**

Participants perceived that promoting effective AMS was a priority for South African primary healthcare. However, it was clear that there are many hurdles to overcome working in settings that are relatively resource-poor. Prescribing guidelines needed to be harmonised. Participants felt that staff training on the principles of AMS should be mandatory. Research was urgently needed to better understand their community’s understanding of antibiotic use and AMS, and to champion outreach projects in the community.

**Conclusion:**

Important stakeholder perspectives in the community were highlighted to promote a multidisciplinary approach to AMS initiatives in primary care. These will need to be addressed to optimise antibiotic prescribing in the community.

## Background

Antibiotic resistance is an important societal health issue requiring global yet context-specific solutions. Overprescribing and inappropriate prescribing of antibiotics are the principal modifiable drivers of antibiotic resistance.^1^ Most antibiotics (75%) are prescribed in the community for common infections, and antibiotic-resistant infections make patients sicker for longer in the community.^2^ Promoting evidence-based antibiotic prescribing through antibiotic stewardship programmes is critical and requires a coordinated multidisciplinary effort.^3^

South Africa has shown much progress with major shifts in policy towards combatting antibiotic resistance. The country has a blueprint for the steps to be taken by healthcare practitioners and others to enact antibiotic stewardship at all levels of healthcare^4^ and practical steps to address the governance framework needed at each level of the health system.^5^ This will become increasingly important in the transition to the country’s anticipated National Health Insurance (NHI) where pharmacists are likely to expand their role for primary healthcare services to increase access.^6^

Pharmacists and clinicians are being increasingly engaged in antibiotic stewardship partnerships to improve clinicians’ antibiotic prescribing. Antibiotic stewardship programmes aim to promote evidence-based antibiotic prescribing and typically involve educational programmes, the implementation of evidence-based guidelines, and audit and feedback for prescribers.^7^

Although the ‘practitioner–pharmacist’ collaboration model exists, this is more commonly found in hospitals than in community-based primary care. In hospitals, pharmacists play an active role in improving the appropriateness of antibiotic prescribing practice through the provision of expert advice, education and training, liaison with regards to formulary, the provision of resistance data, raising awareness of guideline adherence and policy-guided antibiotic prescribing.^8^ In primary care, a recent review showed that such partnerships can be effective in decreasing the antibiotic prescribing rate (odds ratio [OR] 0.86, 95% confidence interval [CI] [0.78–0.95], 8 trials) and increasing guideline-adherent antibiotic prescribing (OR 1.96, 95% CI [1.56–2.45], 10 trials).^9^ Included studies, however, were all conducted in high-income countries (HICs). None were conducted in Africa. Yet, evidence generated in HICs is not always applicable because of differences in the social determinants of health.^10^

We therefore set out to explore the feasibility of a pharmacist–prescriber network in publicly funded primary care facilities to optimise antibiotic prescribing by frontline clinicians. We specifically wanted to know how and where such pharmacist–prescriber interventions might work in primary care clinics in the greater Cape Metro region and to identify the opportunities and barriers of good stewardship at primary care facilities in the region.

## Methods

We advertised and ran a local AMS workshop, held in October 2019 in Bellville, Cape Town. We invited any practising frontline pharmacists, nurses involved in prescribing, and doctors working in Community Health Centres (CHCs) or Community Day Centres (CDCs). Health professionals working solely in hospitals were excluded. We did not pre-specify a participant’s level of seniority or experience, but rather took an inclusive ‘bottom-up’ approach, focussing on a participant’s unique perspective on prescribing at their CHC or CDC, and their potential to contribute meaningfully to the workshop.

Participants were divided into smaller groups to facilitate a balanced discussion of four topics (see Box 1) and then fed back to the wider group for further debate. The four topics were modelled on previous workshops conducted internationally and adapted for the local setting (Philip Howard and Elizabeth Beech, pers. comm., September 2019). Key suggestions for interventions and opportunities to improve antibiotic prescribing within the primary care setting were discussed.

BOX 1 Group workshop questions on community antimicrobial stewardship.

- What stewardship strategies can be implemented at primary care facilities? Give examples.
- What are the barriers of stewardship at primary care level and how can they be overcome?
- What are the enablers of stewardship in primary care facilities?
- What social (local community changes) and behavioural aspects should be addressed to advance stewardship?

*Source:* Reproduced and adapted with permission from Professor Philip Howard, British Society for Antimicrobial Chemotherapy.

### Ethical consideration

We confirm that ethical clearance was not needed/required for this study.

## Results

**FIGURE 1:**
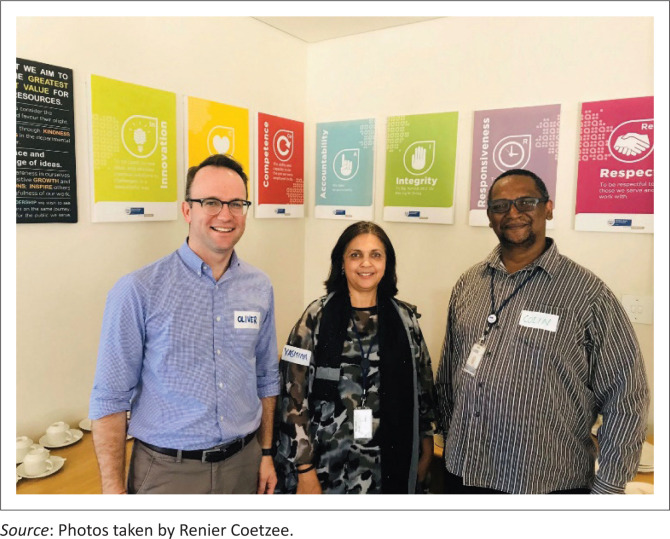
Local collaborators from the Western Cape Department of Health.

**FIGURE 2:**
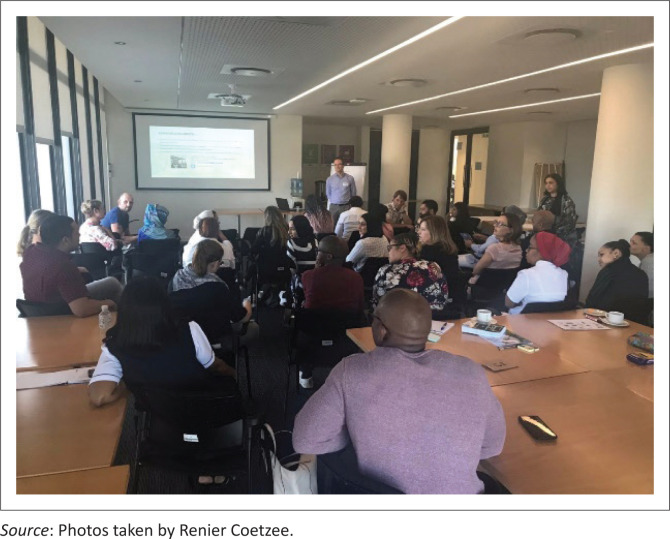
Participants engaging in a discussion at the antimicrobial stewardship workshop.

Thirty-three participants contributed to the workshop (9 doctors, 5 nurses, 18 pharmacists and 1 pharmacist assistant) (see Figure 1 and Figure 2). Participants thought that AMS was a priority for South African primary healthcare. They voiced their frustration of the difficulties of applying good AMS principles in the context of resource-poor settings. A high staff turnover, work absenteeism, power cuts, stock-outs and an increasing clinical workload made AMS less of a priority.

The participants valued the use of readily available local and national prescribing guidelines. However, they pointed out that there were often conflicting prescribing regimens. Local communication circulars were often at odds with guidelines and some clinics were using guidelines that had been superseded. There were also concerns raised that private pharmacists at the community pharmacy level were often overlooked in current AMS initiatives yet have an important role to play.

Although a timely audit and feedback were important, participants thought that time constraints, poor record-keeping and inadequate dissemination to the entire team made them question the value of prescribing audits. Participants perceived that the attitudes of patients towards prudent antibiotic use was at times unhelpful. This was compounded by inadequate time to discuss a non-antibiotic strategy, provide relevant health promotion (e.g. hand hygiene) or safety-netting of antibiotic use. Participants acknowledged that strong local leadership and good clinical governance were key. They suggested that all patient-facing clinic staff in the community should be required to have standard training in AMS.

Participants wanted all clinic staff to show a unified front in applying an evidence-based approach to antibiotic prescribing and build patient trust in healthcare facilities. Thus, research was urgently needed to better understand their community’s understanding of antibiotic use and AMS. They felt that community healthcare workers might be best placed to champion AMS outreach projects. They also wanted additional initiatives on how best to engage patients about antibiotic use through relevant promotional media and health campaigns tailored to the local community.

## Discussion

This workshop provided critical perspectives from stakeholders in publicly funded healthcare facilities to promote a multidisciplinary approach to AMS initiatives in primary care. We found that despite the enormous hurdles to overcome in the public health sector, there was an awareness and collaborative spirit to make AMS a priority.

Clinically, our findings support the need for clinics to ensure that the latest prescribing guidelines are being used and to communicate this to all staff – for example, setting up a suitable information-sharing platform (work WhatsApp group). All staff need to demonstrate their competencies in AMS by completing freely available AMS training modules (e.g. www.fidssa.co.za/SAASP/Edu_Material). This should also be extended to those health professionals still in training and private community pharmacies.

For policymakers, local and national prescribing guidelines need to be congruent with each other. They should also consider developing guidelines for common infection syndromes where antibiotics might not provide any additional benefit. Engaging patients in the discussion about antibiotic use, its harms and benefits for common infection syndromes and so forth needs to be incorporated into future health campaigns, bringing relevant information to the local community. Patients should receive clear information, ideally reinforced with leaflets or text messages, about the likely duration of symptoms, self-care, and the likely benefits and harms of antibiotics.

Future research should focus on establishing studies to prospectively collect antibiotic prescribing data in primary care. This would require novel collaborations between pharmacists and prescribers to collaborate and collect prescribing data and link these to clinical indications. Currently, these data are retrospective and not linked to clinical indications. This is a major evidence gap but essential to describe the problems experienced about access to, and excess of, antibiotics for acute infection syndromes. Such descriptive studies are key to informing larger trials that can support the improved use of antibiotics.

## Conclusion

Our multidisciplinary workshop raised important stakeholder perspectives on potential AMS initiatives in primary care. Strategies that can optimise antibiotics can help to provide consistency in healthcare delivery, reduce inequality in healthcare provision, and ensure the continued effectiveness of antibiotics for common infections.
